# Icosahedra clustering and short range order in Ni-Nb-Zr amorphous membranes

**DOI:** 10.1038/s41598-018-24433-9

**Published:** 2018-04-17

**Authors:** S. Sarker, D. Isheim, G. King, Q. An, D. Chandra, S. I. Morozov, K. Page, J. N. Wermer, D. N. Seidman, M. Dolan

**Affiliations:** 1 Departments of Chemical and Materials Engineering, University of Nevada, Reno, Nevada 89557 USA; 2 Department of Materials Science and Engineering, Center for Atom Probe Tomography, Northwestern University, 2220 Campus Dr., Evanston, IL 60028 USA; 3 Materials Science and Technology Division, Los Alamos National Laboratory, Los Alamos, NM 87545 USA; 4 Department of Computer Simulation and Nanotechnology, South Ural State University, Chelyabinsk, 454080 Russia; 5 Chemical and Engineering Materials Division, Oak Ridge National Laboratory, Oak Ridge, TN 37831 USA; 6 CSIRO, Division of Energy Technology, Kenmore, QLD 4069 Australia

## Abstract

Crystalline Pd/Pd-Ag membranes are widely used for hydrogen separation from CO_2_ and other gases in power generation applications. To substitute these high cost noble metal alloy membranes, the Ni-Nb-Zr amorphous alloys are being developed that exhibit relatively high permeability of hydrogen between 200–400 °C. Atom probe tomography (APT) experiments performed on these ribbons revealed nm-scale Nb-rich and Zr-rich regions (clusters) embedded in a ternary matrix, indicating phase separation within the Ni-Nb-Zr amorphous alloy. Density functional theory (DFT) simulations have predicted that these clusters are composed of icosahedral coordination polyhedra. The interatomic distances and correlation lengths of the short range order of these alloys were determined by neutron total scattering which match well with our DFT based molecular dynamics (DFT-MD) simulations.

## Introduction

Ni-based amorphous alloys have been developed to replace crystalline Pd/Pd-23Ag (23% Ag) noble metal alloy membranes which are commercially available for hydrogen separation from CO_2_ and other gases in power generation applications. Hara *et al*.^1^ proposed using amorphous alloy membranes of Ni_64_Zr_36_ for separating hydrogen from CO_2_. Since then many permeation studies were performed to understand permeation behavior of Ni-based amorphous alloys; these are discussed in a review paper^2^. In spite of the lack of long range order, these amorphous alloys show high hydrogen permeability, very good electrical transport properties, and under certain conditions show superconductivity^2,3^.

In addition, metallic glasses have outstanding mechanical properties such as extreme strength and hardness, excellent formability in the supercooled liquid region, large elastic strain limit, and high wear and corrosion resistance compared with crystalline counterpart due to a lack of crystal defects and grain boundaries discussed in the literatures^4–7^. Ni based metallic glass showed 6.6% super elastic stain limit^8^. These metallic glasses possess promising applications for biomedical materials^4^, light-weight structural materials^7^, coating purposes^7^ and magnetic core inductor^9^, however, due to their brittleness, they are not widely used for commercial applications.

Therefore, it is very important to understand the nature of internal atomic arrangement of these metallic glasses. Several studies have been performed^10–12^ in the past decade to understand the atomic arrangement of (Ni_0.60_Nb_0.40_)_70_Zr_30_ glassy ribbons. There are reports of icosahedral structure formation inside the ribbon^3,11,13^, although fundamental understanding of the cluster formation, structural configuration, internal atomic arrangement and chemical homogeneity in this amorphous ribbon are still lacking. Also, phase separation (chemical heterogeneity) in Ni-Nb-Zr amorphous membranes, without prior annealing to crystallization and cluster effects on glass formability, mechanical, electrical and dynamic properties have not been reported earlier to the best of our knowledge.

To address these issues we performed a combination of atom probe tomography (APT) and neutron scattering (HIPD) experiments coupled with the density functional theory (DFT) based molecular dynamics (MD) simulations on the membrane alloy ribbons of (Ni_0.60_Nb_0.40_)_70_Zr_30_. The APT experiments were performed in order to understand the chemical heterogeneity and local chemical composition of these alloys and to determine the possible existence of clusters and their average sizes and compositions. These APT data allowed us to reconstruct three dimensional structures revealing Nb-rich and Zr-rich clusters embedded in a ternary matrix whose compositions deviated from the nominal overall composition of the membrane. Here we used phase separation to represent the emergence of the Nb-rich and Zr-rich clusters embedded in the glass matrix (chemical heterogeneity). Next, we performed a neutron total scattering experiment that gave interatomic distances between all types of atom-atom pairs. Finally, the DFT-MD simulations provided detailed local cluster information of Nb-rich and Zr-rich clusters in the Ni-rich matrix. In addition, the radial distribution functions derived from the DFT-MD simulations gave the interatomic distance between atom pairs corroborated very well with those of the experimentally determined values. In this study, we present the local atomic order of the (Ni_0.60_Nb_0.40_)_70_Zr_30_ glassy alloy membrane ribbon and discuss cluster formation obtained from the APT experiments and interatomic distances between atoms measured by neutron scattering experiments and DFT simulations.

## Results and Discussion

### Atom Probe Tomography

**Figure 1:**
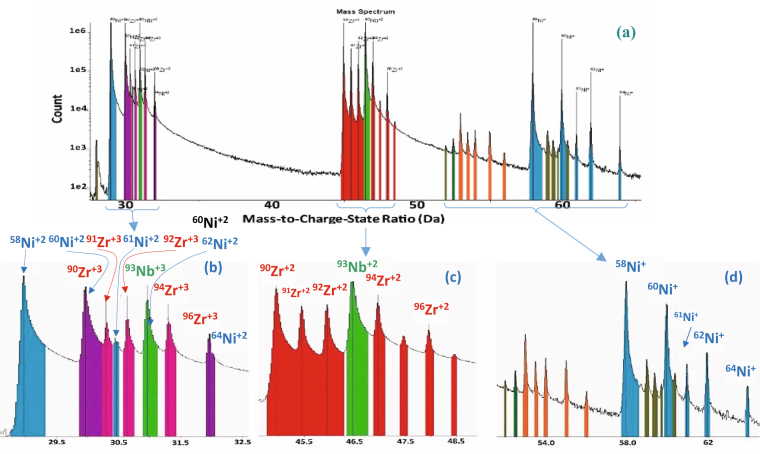
(**a**) The overall mass spectrum binned in 0.02 amu in the mass reange 27–66 amu for the area shown in figure, (**b**) expanded region of the spectra in 0.02 bins 27–66 amu, (**c**) expanded region of the spectra in 0.02 bins 43.5–50 amu, (**d**) expanded region of the spectra in 0.02 bins 50–66 amu.

**Figure 2:**
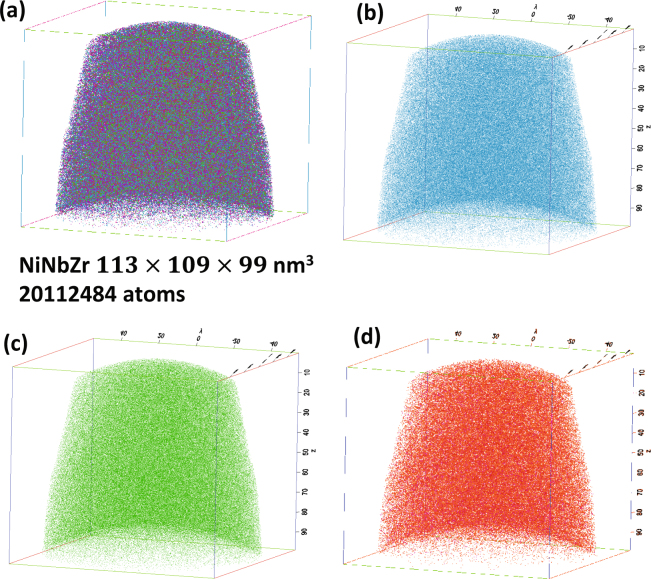
Atom probe 3D reconstruction of a portion of the (Ni_60_Nb_40_) _70_Zr_30_ needle shaped specimen showing (**a**) ~20.1 million atoms of Ni, Nb and Zr, and other small impurities in a section 113 × 109 × 99 nm^3^ volume, (**b**) Ni atoms (isolated), (**c**) Nb atoms (isolated), and Zr atoms (isolated).

Atom Probe Tomography (APT) was performed on the needle shaped specimen of (Ni_0_._60_Nb_0.40_)_70_Zr_30_ obtained from the thin alloy membrane. The alloying elements Ni, Nb, Zr and their respective isotopes are detected by time-of-flight mass spectrometry, following field-evaporation of the atoms activated by the laser pulses. The mass spectra show the isotopic peaks for Ni^+^, Ni^2+^, Zr^2+^. Zr^3+^, Nb^2+^, and Nb^3+^ in the range ~28 to 65 mass-to-charge-state ratio (in “amu” units). For clarity, we expanded the three sections where the peaks are located as shown in Fig. 1. The expanded range of 28.5–32.5 amu shows the peaks of Ni^2+^ (isotopes 58, 60, 61, 62, and 64) and Zr^3+^ (isotopes 90, 91, 92, 94, and 96) and Nb^3+^ (isotope 93) in Fig. 1(b). There are isobaric overlaps between the peaks for ^60^Ni^2+^ and ^90^Zr^3+^, ^64^Ni^2+^ and ^96^Zr^3+^, and ^62^Ni^2+^ and ^93^Nb^3+^. In the range of 44.5–49 amu, all isotopic peaks of Zr^2+^(90, 91, 92, 94, and 96) and one peak for ^93^Nb^2+^ are observed in Fig. 1(c). The peaks of Ni^+^(58, 60, 61, 62, and 64), and other Hf, Ta, O (complex molecular ions (ZrO)^2+^) and C (NbC)^2+^ impurities are observed in the mass range 52–64.5 amu, Fig. 1(d). For convenience, a complete list of the isotope mass numbers, atomic mass, and natural isotope abundances and the typical charge states for the elements Ni, Nb and Zr are shown in Table 1. The isobaric overlaps are corrected to obtain the overall compositions with a deconvolution calculation based on the respective isotopic abundances. While this correction can be done for a collection of ions with sufficient counting statistic, it cannot be applied for determining the chemical identity of individual ions, this limits the capability for atomically resolving individual coordination polyhedra, in addition to the 50% detection efficiency limit mentioned above. A three dimensional APT reconstruction of the glassy alloy (Ni_0_._60_Nb_0.40_)_70_Zr_30_ shows the distribution of Ni, Nb, and Zr atoms in a 113 nm × 109 nm × 99 nm volume in Fig. 2(a). Figure 2(b–d) show the distribution of individual elements Ni (blue), Nb (Green) and Zr (Red) atoms in this reconstruction volume. The nominal average composition of the amorphous matrix of this ribbon is Ni = 42 at.%, Nb = 28 at.% and Zr = 30 at.%. Even though the atomic distribution appears to be fairly uniform to the eye, the local composition needs to be characterized with respect to potential (i) local nanometer-scale compositional variations related to decomposition of multiple amorphous phases, and (ii) we seek information about the atomic arrangement, short range order (SRO), and polyhedra.

**Table 1:** Composition of Phase I (Nb-rich cluster), Phase II (Zr-rich cluster), and overall composition of the alloy from the atom probe tomography studies.

Phase Name	Abbreviation	Ni	Elemental Concentration (at.%)				
			Statistical error	Nb	Statistical error	Zr	Statistical error
*Overall Nominal*	Ni_42_Nb_28_Zr_30_	42	—	28	—	30	—
*Overall measured by APT*	Ni_42_Nb_26_Zr_32_	41.97	±0.07	26.05	±0.03	31.98	±0.07
*Phase I (Nb-rich cluster)*	Ni_32_Nb_46_Zr_22_	31.8	±0.6	46.5	±0.4	21.7	±0.7
*Phase II (Zr-rich cluster)*	Ni_39_Nb_18_Zr_44_	38.6	±0.8	17.9	±0.3	43.45	±1.1

**Figure 3:**
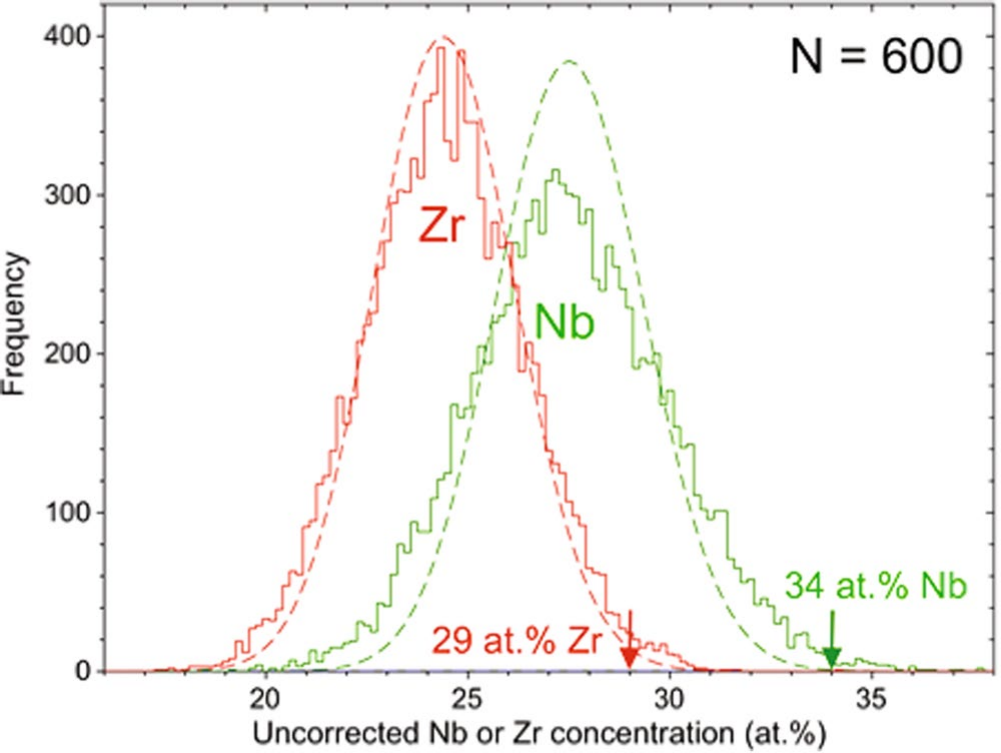
Concentration frequency distribution analysis of a subvolume of the APT reconstruction of the (Ni_0_._60_Nb_0.40_) _70_Zr_30_ melt-spun ribbon.

The presence of compositional variations or inhomogeneities is shown in the Fig. 3. The graph shows the distributions of concentrations for Zr and Nb determined from small blocks containing N = 600 atoms. The comparison with the binomial distribution, dashed lines, representing a uniform atomic distribution, reveals that both the measured distributions are significantly broader than the uniform distribution with significant tails to higher Zr concentrations above 29 at.% Zr and higher Nb concentration above 34 at.%. The significance of the concentration tails and broadening relative to a uniform alloy was confirmed with a chi-square test. The arrows in Fig. 3 delineate compositions, 29 at.% Zr or 34 at.% Nb, above which the majority of blocks with concentrations above these limits are non-random. We use these concentration thresholds in the following for isolating the Zr or Nb-rich regions, their spatial distribution, and perform a detailed on positional analysis.

**Figure 4:**
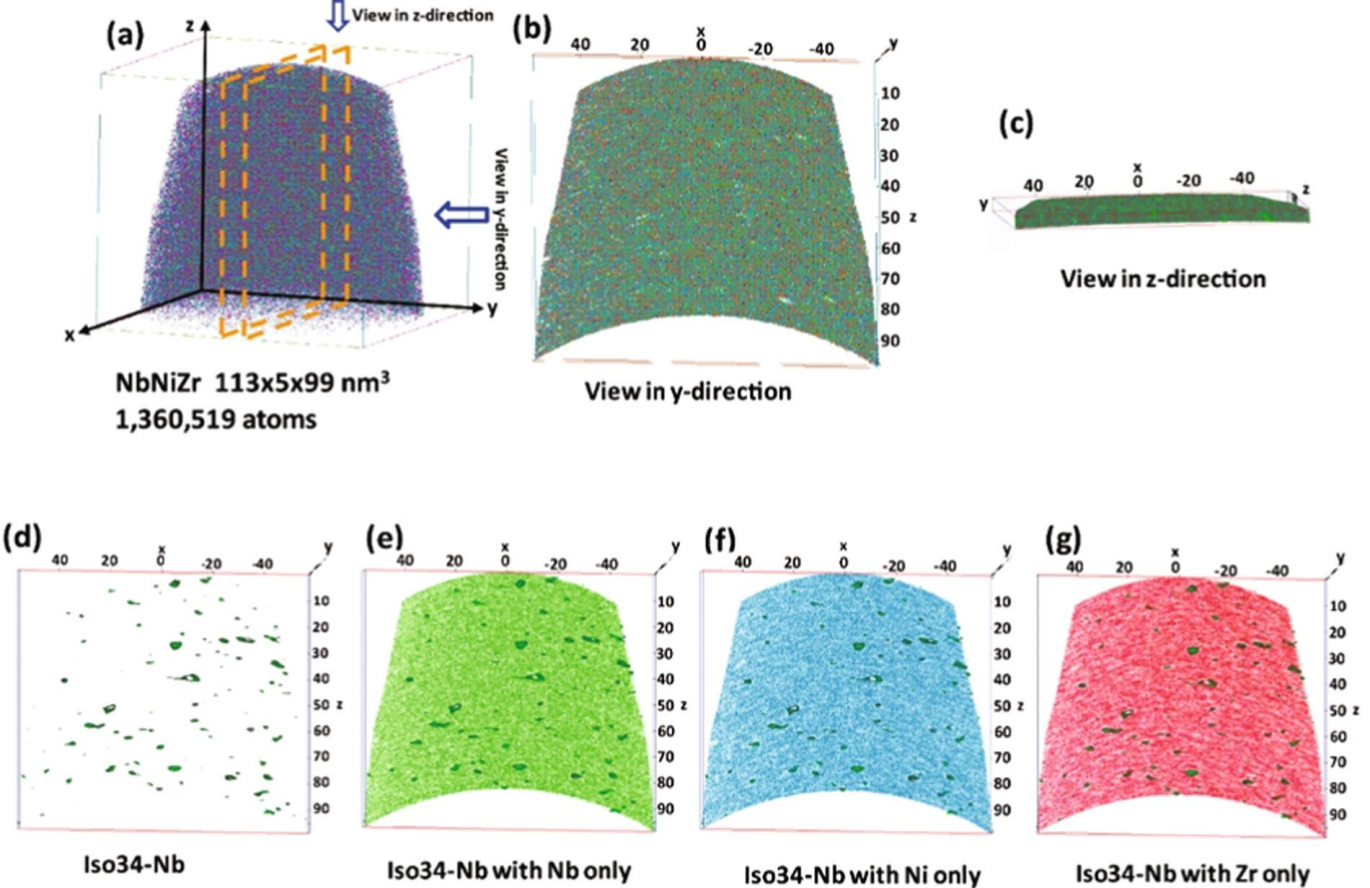
(**a**) Atom probe 3D reconstruction of a portion of the (Ni_0.60_Nb_0.40_)_70_Zr_30_ sample in a cropped volume of 113 × 5 × 99 nm^3^ showing all the Ni, Nb, and Zr atoms, (**b**) view in *y*-direction, (**c**) view in *z*-direction, (**d**) Iso34-Nb, (**e**) Iso34-Nb with Nb atoms (isolated), (**f**) Iso34-Nb with Ni atoms (isolated), and (**g**) Iso34-Nb with Zr atoms (isolated).

**Figure 5:**
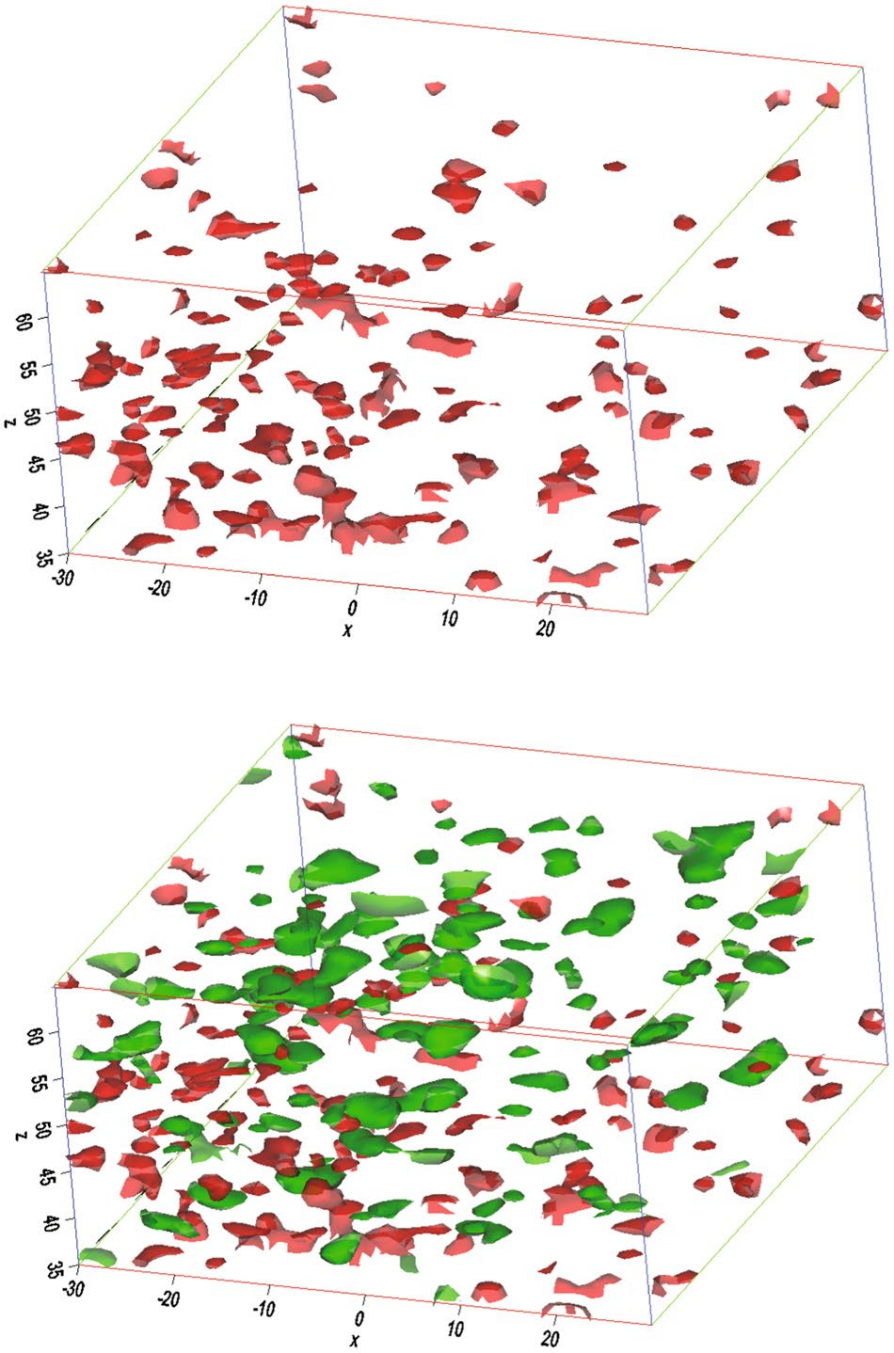
(**a**) Zirconium rich regions delineated by a 29 at.% isoconcentration surface (red color), (**b**) niobium rich regions (34 at.% isoconcentration surface, green color). The cluster size of niobium is comparatively bigger than the cluster size of zirconium.

We first revisit the spatial distribution of Zr, Nb, and Ni in a small volume slice of 113 × 5 × 99 nm^3^ selected from the center of the original volume of 113 × 109 × 99 nm^3^, as shown by dotted lines Fig. 4(a). The atomic reconstruction and 34 at.% Nb isoconcentration surfaces revealed inhomogeneous amorphous structure with Nb-rich clusters embedded in a Ni-rich matrix. Sections in the *y* and *z* directions can be seen in Fig. 4(b) and (c). We will first discuss the detection of the Nb-rich clusters in the matrix. The matrix has 28 at.% Nb, but varying the concentration greater or less than 28 at.% we observe Nb-rich clusters of different composition than the nominal ones (Fig. 4(d)); other atoms 113 × 109 × 99 nm^3^ of 34% Nb, that shows the same clusters as seen in Fig. 4(d), but superimposed with lower concentration Nb-atoms. The position of the clusters remains the same when these 34% Nb atoms are superimposed with the Ni (Fig. 4(f)), and Zr (Fig. 4(g)) atoms. We designate this Nb-rich cluster as *Phase I*. In a similar manner we determined Zr rich (*Phase II*) in the matrix by using a 29 at.% Zr isoconcentration surface, Fig. 5(a). These results clearly suggest phase separation even at room temperature in the amorphous alloy (Ni_0.60_Nb_0.40_)_70_Zr_30_ ribbon with separate Nb and Zr-rich clusters, respectively. In Fig. 4(e) we show Nb atoms above and below the threshold iso-concentration. The distributions of isolated Zr-rich clusters (in red color) in a small volume are shown in Fig. 5(a); the dots representing other atoms are left out for clarity. The spatial distribution of both Nb- and Zr rich regions in the (Ni_0.60_Nb_0.40_)_70_Zr_30_ ribbon are shown in Fig. 5(b). The average composition of the ribbon as measured by the APT is Ni = 41.89 at.%, Nb = 26.14 at.%, Zr = 31.97 at.% which is very close to the nominal alloy composition of Ni = 42 at.%, Nb = 28 at.%, Zr = 30 at.% of the melt spun ribbon. Table 2 shows the composition of Nb and Zr enriched cluster which are distinctly different than the nominal composition of the alloy. We estimated the cluster size of Zr-rich region as ~2–3 nm, and ~4–5 nm in the Nb-rich region from Fig. 5.

**Table 2:** Stable isotopes of and field-evaporation properties of Ni, Zr, and Nb for time-of-flight mass spectrometry in atom-probe tomography (Bold indicate most probable Charged State).

Element	Ni			Zr			Nb		
	Atom-nm^3^	At. wt.	At. No	Atom-nm^3^	At. wt.	At. No	Atom-nm^3^	At. wt.	At. No
	91.4	58.693	28	43.0	91.224	40	64.2	92.906	41
Charge State	**+**	**++**	**+++**	**+**	**++**	**+++**	**+**	**++**	**+++**
Evaporator Field (V/nm)	**35**	36	65	**56**	28	35	**74**	37	45
**Sr. No**.	*Isotope Mass No.	**Isotope (At. %)**	**Atomic mass (Da)**	*Isotope Mass No.	**Isotope (At. %)**	**Atomic mass (Da)**	*Isotope Mass No.	**Isotope (At. %)**	**Atomic mass (Da)**
1	58*	57.935	68.077	90*	89.905	51.45	93*	92.906	100
2	60*	59.931	26.223	91*	90.906	11.22			
3	61*	60.931	1.140	92*	91.905	17.15			
4	62*	61.928	3.635	94*	93.906	17.38			
5	64*	63.928	0.926	96*	95.908	2.8			

So far, we have identified the presence of Zr- and Nb- rich clusters in the amorphous matrix, but the detailed atomic structure of these clusters and actual short range order remain to be determined. We established characteristic nearest neighbor (NN) distances of Ni-Ni, Nb-Nb, and Zr-Zr in the ribbon by APT, using the algorithm implemented in the IVAS3.6.12 software package, similar to the approach described by Shariq *et al*.^13,14^ for measuring the nearest neighbor distance in ternary amorphous alloys by APT. This approach assumes that the limited detection efficiency and spatial resolution in APT results in a broadening of the NN distance distribution, but not in a shift of the most likely NN distance, preserving the non-random information related to local short-range order in a coordination polyhedron, so that the most frequent distance in the distribution can be identified with the actual next-neighbor pair distance, in our case the Ni-Ni, Nb-Nb, and Zr-Zr next neighbor pair distances. This approach can be justified if, on average, at least one next neighbor of each constituent element is preserved in the APT reconstruction. Given a 50% detection efficiency of the APT detector, and 12–14 next neighbor atoms in a typical coordination polyhedron, 6-7 next neighbors of each atom are preserved, on average, in the APT reconstruction. Since at least one atom of each elemental species is needed for resolving the atomic pairings of the coordination polyhedron, a minimum average atomic concentration of 14–17 at.% is required which is the case for the amorphous alloys studied by Shariq *et al*.^13,14^. In our case, however, the isobaric overlaps between some of the elements and charge states in the mass spectrum, see Fig. 1 and Table 1, further reduce the amount of usable information. Specifically, to unambiguously identify Ni and Zr, only the isotopes 58 for Ni and the isotopes 91, 92, and 94 for Zr can be used, representing 68.077% of Ni and 45.75% of Zr, see Table 1. We neglect the small contribution of ^62^Ni^++^ to the large peak for ^93^Nb^3+^ at 31 amu. Given the atomic elemental concentrations listed in Table 2, this results in an isotopic concentration for ^58^Ni of 28.6 at%, and only 14.6% for Zr, which is close to the very limit of where the assumption of having at least one next neighbor atom present in the reconstruction, on average, breaks down, which will result in an increased error of the measurement. The loss of structural information relating to the short-range order for lower elemental or isotopic concentrations will cause a shift of the peak in the next-neighbor distribution, in addition to the broadening mentioned above, as discussed by Philippe *et al*.^15^ for the very different case of randomly distributed solute atoms in a dilute solution. For the sake of comparison, we determined the next-neighbor distances for the Ni-Ni, Nb-Nb, and Zr-Zr atom pairs in the amorphous (Ni_0.60_Nb_0.40_)_70_Zr_30_ alloy with the approach outlined by Shariq *et al*.^13,14^, using the most likely distance in the next-neighbor distribution, and the resulting distances are given in Table 3.

**Table 3:** Next-neighbor distances for Ni-Ni, Nb-Nb, and Zr-Zr pairs measured by atom-probe tomography.

Element Pair	Ni-Ni	Nb-Nb	Zr-Zr
Interatomic Next-neighbor distance measured by APT (nm)	~2.53	~2.65	~3.05

In the literature, Kelton *et al*.^16^ reported experimental demonstration of Frank hypothesis in Ti_39.5_Zr_39.5_Ni_21_ alloy in undercooled liquids local structure that contained a significant degree of short-range order (SRO) icosahedra, with no long-range periodicity. Many others reported metallic glass systems have a significant degree of order within the amorphous matrix^15,16^. Icosahedral short range order has been found in various metallic glasses and its fraction has been used as a characteristic indicator of the formation of metallic glasses^17,18^.

There are examples of Zr-based amorphous alloys that showed phase separation and nanoscale icosahedral clusters formation in a single amorphous phase by APT close to glass transition temperature^19^. The DFT-MD simulation reveals a similar type of icosahedral structure appearing in our ribbon at room temperature^3,11,12,19^. In another paper, Hono *et al*.^20^ obtained three dimensional APT data in which they observed clustering of Cu just prior to primary crystallization. Shariq and Mattern’s APT study^21^ revealed phase separation in Ni_66_Nb_17_Y_17_ alloy, where the Y-rich phase is depleted of Nb and vice-versa. They also found two interconnected amorphous phases similar to what we observed in our Ni-Nb-Zr alloy. There are reports of amorphous thin films in the immiscible copper–niobium system in which phase separation was observed even at low temperatures, 200 °C^22^. Mattern *et al*.^23^ studied as-quenched Cu_46_Zr_47−x_Al_7_Gd_x_ metallic glasses and found that the cluster formation was dependent on the Gd content in the alloy; this formation is favorable at higher temperatures due to pre-crystallization nucleation and growth. All the above mentioned literature and our APT data suggested that phase separation might be a common behavior in the majority of the amorphous materials. Our ribbon is stable for several weeks during the permeation experiments, suggesting that the phase separation occurred during quenching in the melt spinning process.

In 2009, Oji *et al*.^11,12^ reported icosahedral structure in (Ni_0.60_Nb_0.40_)_70_Zr_30_ and (Ni_0.60_Nb_0.40_)_60_Zr_40_ alloys using XAFS data and other computational models. Further studies by Fukuhara and Inoue^3^ using XANES confirmed that these glassy alloys have formed icosahedra. Oji *et al*.^12^ proposed two possible icosahedra structure models Zr_6_Ni_6_Nb and Ni_5_Nb_3_Zr_5_ for the ternary alloys_._ A distorted icosahedral structure of Ni_5_Nb_3_Zr_5_ type was reported for the alloy of (Ni_0.60_Nb_0.40_)_60_Zr_40_ by molecular dynamics calculations^3,11^. In order to understand detailed local cluster structures inside these Ni-based amorphous ribbons by considering the experimental APT data, DFT-MD simulation were performed. The DFT-MD simulations cannot predict the whole experimental samples because of the limited size scale to hundreds of atoms. However, it can provide physical insight on the local heterogeneous cluster structures composed by a hundred of atoms. The DFT-MD results suggest that the amorphous structure consists of different types of icosahedra shown in the Table 3; these icosahedra consists of 12 atoms (Voronoi index (0 0 12 0)) and most of them are Ni and Nb centered. The Nb- and Zr-rich clusters in the alloys have different icosahedral chemical formulas as expected shown in Table 4. The DFT simulation reveals the average size of single icosahedron is ~0.56 nm.

**Table 4:** Different icosahedra configurations for Nb-rich (Ni_41_Nb_59_Zr_28_) and Zr-rich (Ni_49_Nb_23_Zr_56_) with centered atom in parenthesis calculated by DFT-MD.

Icosahedron	Ni_41_Nb_59_Zr_28_	Ni_49_Nb_23_Zr_56_
1	Ni_2_Nb_7_Zr_3_ (Zr)	Ni_3_Nb_4_Zr_5_ (Nb)
2	Ni_4_Nb_4_Zr_4_ (Nb)	Ni_2_Nb_4_Zr_6_ (Nb)
3	Ni_4_Nb_6_Zr_2_ (Nb)	Ni_4_Nb_2_Zr_6_ (Ni)
4	Ni_2_Nb_5_Zr_5_ (Nb)	
5	Ni_1_Nb_7_Zr_4_ (Ni)	
6	Ni_3_Nb_6_Zr_3_ (Ni)	
7	Ni_4_Nb_5_Zr_3_ (Ni)	
Average over icosahedra	Ni_16_Nb_30_Zr_18_	Ni_9_Nb_10_Zr_17_
Average over all	Ni_31_Nb_49_Zr_26_	Ni_33_Nb_18_Zr_48_

**Figure 6:**
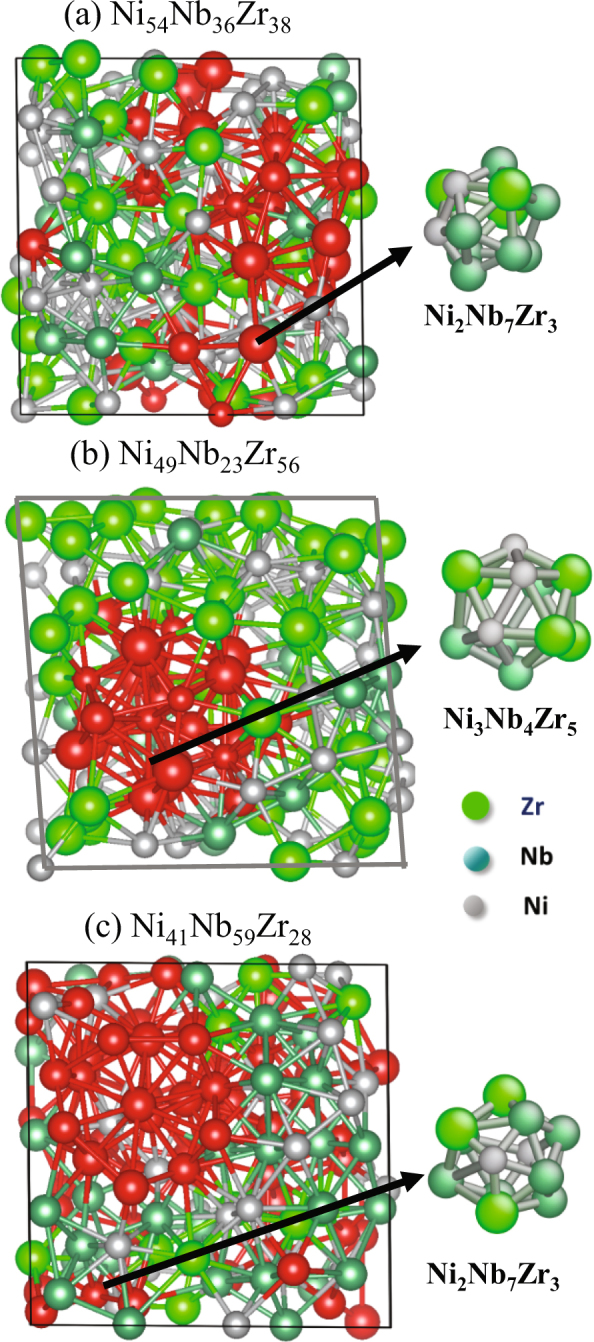
Snapshots of various ternary metallic glasses which are formed during the fast quenching from high temperature of 3000 K with the cooling rate of 1.08 × 10^14^ s^−1^. The atoms within icosahedral clusters are represented by red balls in the periodic simulation box. The Zr, Nb and Ni atoms are represented by green, cyan and grey balls, respectively.

The Fig. 6 showed snapshot of (a) nominal composition (Ni_54_Nb_36_Zr_38_), (b) Zr-rich region (Ni_49_Nb_23_Zr_56_) and (c) Nb-rich region (Ni_41_Nb_59_Zr_28_) where Ni, Nb and Zr atoms are represented by grey, cyan and green balls respectively. The atoms with icosahedra clusters represented by red atoms revealed that the Nb rich cluster is composed of 7 icosahedra (~3.9 nm) and Zr- rich cluster is composed of 3 icosahedra (~1.7 nm) which are similar to the experimental data shown in Fig. 5. The cluster typically observed in small region composed of hundred atoms and it is randomly arranged throughout the glass. The APT experimental and DFT-MD simulation shows that the cluster sizes in these metallic glasses between 2–5 nm as a function of different alloying additions^23^. Fujima *et al*.^24^ reported 8 icosahedra of Ni_5_Nb_3_Zr_5_ single unit in (Ni_0.60_Nb_0.40_)_60_Zr_40_ alloy using first principle calculation; according to their calculation local icosahedra structure are stable in the amorphous phase at room temperature. Although we observed the icosahedral clusters at room temperature in DFT-MD simulations, we expected that the phase separation in experiments happens at supercooled liquid where not many icosahedral clusters are formed^18^.

These interconnected icosahedra affect both structural and dynamical properties of metallic glasses^25,26^. Wen *et al*.^25^ performed a series of MD simulations which reveal correlation of glass forming ability (GFA) and icosahedral clusters. We proposed that the Nb and Zr interconnected icosahedral clusters increase glass forming ability^27^ as they lower the atomic packing and minimize the energy, thus stabilize the amorphous structure^28^.

During solidification, the atoms which help to form medium range icosahedra clusters play a crucial role in formation of amorphous alloy. Yang *et al*.^29^ study reveals these interconnected icosahedra progressively increase with decreasing temperature. The APT and DFT-MD simulation reveals tendency to form favorable Nb-rich cluster than Zr-rich cluster. These Nb-rich interconnected icosahedra cause more compact and higher density (7.94 g/cm^3^) structure than Zr-rich (7.46 g/cm^3^) region. Because of these interconnected Nb icosahedral clusters, it is difficult for supercooled liquid to form another metastable basin^26^ which internally restrain crystallization; the system is completely amorphous even at room temperature. Fujima *et al*.^24^ also proposed similar effect of Nb atoms on these amorphous materials.

The strength of the metallic glass increases as number of full icosahedra increase which causes more resistance to the plastic flow of the alloys^27,30^. This plastic flow can be initiated by decreasing full icosahedra of Nb and Zr- rich regions and increasing homogeneity of clusters throughout the matrix. The APT and DFT results showed Nb-rich region is composed of 7 icosahedra, thus, exhibit excellent structural and thermal stability. Non-isotherm DSC experiment also showed crystallization temperature and the thermal stability increases with an addition of Nb in Ni-ETM alloy^31^.

The studies on the Ni-Nb-Zr alloy with addition of hydrogen by Fukuhara *et al*.^32^ suggested sub-nanometersized two different icosahedra cluster configurations that an play important role in the electronic transport behavior of the alloy. Our APT study reveals these clusters are distributed throughout the matrix shown in the Fig. 4, therefore electron travelling through the two different icosahedra is comparatively easier and provides sufficient justification for coulomb oscillation, ballistic transport and superconductivity.

Dynamic heterogeneity such as β-relaxation process is also affected by nano-scale heterogeneity throughout the matrix^33^. The structural relaxation of these amorphous materials depends on the interconnected icosahedra cluster formation. The reason for the relaxation time increases may be due to larger connectivity of icosahedra clusters in Nb-rich and Zr-rich region^26^. In this (Ni_0.6_Nb_0.4_)_70_Zr_30_ metallic glass, the β relaxation time in vacuum is 9780 seconds^34^. The dominant Nb-rich and Zr –rich interconnected full icosahedra are responsible for the dynamic slowdown of this metallic glass^27^. From potential-energy landscape perspective, due to five-fold symmetry of full icosahedra in both Nb and Zr rich regions, the metallic glass has higher configurational transition barrier, thus deep local minima, which causes increase in relaxation time.

In the permeation study, Zr is added to the base alloy Ni_60_Nb_40_ to increase the hydrogen permeation rate. It is possible that the phase separation or cluster formation is enhanced due to the addition of Zr in the Ni-Nb amorphous alloy as the empirical atomic size of zirconium (r_Zr_ = 155 pm) is much larger than of Ni (r_Ni_ = 135 pm) and Nb (r_Nb_ = 145 pm); the more the Zr in the Ni-Nb alloy, greater the tendency for phase separation^35^. This reason also is applicable for the dynamic slowdown of the system.

### Neutron Total Scattering

**Figure 7:**
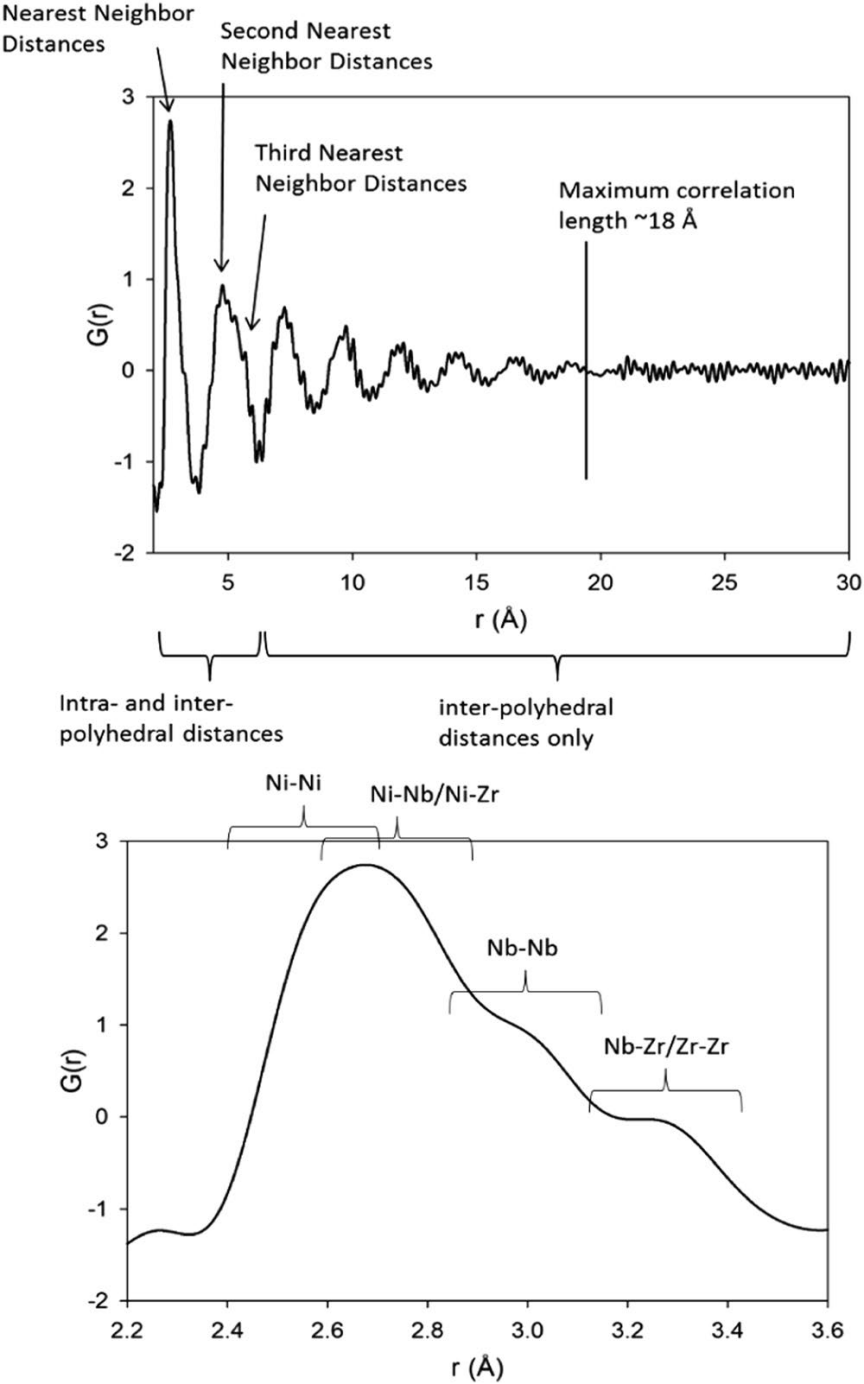
(**a**) Pair distribution function of (Ni_0.60_Nb_0.40_)_70_Zr_30_ showing features up to ~1.8 nm. (**b**) Details of overlapped regions of the first nearest neighbors distances of Ni-Ni, Ni-Nb/Ni-Zr, Nb-Nb, and Nb-Zr/Zr-Zr.

We could not determine the interatomic distance of different pairs of atoms, such as Zr-Ni or Ni-Zr by the APT in the (Ni_0_._60_Nb_0.40_)_70_Zr_30_ alloy with better precision, due to detection efficiencies issues and the isobaric overlaps in the mass spectrum, that effectively further reduce the number of atoms that can be identified unambiguously. We turned to total neutron scattering performed at LANSCE (Los Alamos National Laboratory). The pair distribution function (PDF) data shows the number of atomic pairs separated by a distance of “r” and therefore that serves as an excellent probe of the local structure of disordered materials. The PDF of the (Ni_0.60_Nb_0.40_)_70_Zr_30_ alloy (in Fig. 7a) showed well defined features of the short range order up to ~1.8 nm with no structural correlations at longer length scales. The first peak in the PDF plot represents the nearest neighbor atomic distances and has contributions from interatomic bond distances. This first peak has a main feature at ~0.268 nm and two high-r shoulders. The low-r feature represents bond distances involving Ni and is the most prominent feature because Ni is the most common element and has the strongest neutron scattering length. The Ni-Ni distances are expected to be the shortest, but in our case we observed them to be longer (0.240–0.270 nm) as compared to the Ni-Ni distance of only 0.248 nm in the pure metallic Ni, whereas the average Ni-Ni distance is probably a few hundredths of an Å shorter than the 0.268 peak, there are clearly no atom-atom pairs with average distances as short as 0.248 nm. We did not find any feature at or around 0.248 nm, concluding that there are no regions in this material where Ni is coordinated mostly or entirely by Ni in this ternary alloy. Thus it is expected that the matrix of this ribbon is Ni rich with Nb and Zr atoms. We propose that the Ni-rich matrix with significant number of Nb and Zr atoms randomly distributed embeds the Nb- and Zr rich clusters. All Ni atoms appear to be bonded to significant numbers of Nb or Zr atoms, which slightly elongates the Ni-Ni distances relative to pure Ni. The higher-r part of the main feature can be expected to originate from Ni-Nb and Ni-Zr distances, which are on average slightly longer than 0.268 nm. The first high-r shoulder, is centered at ~0.295 nm, and can be expected to be primarily from Nb-Nb distances. The second shoulder at ~0.324 nm can be assigned to Nb-Zr and Zr-Zr distances.

The second peak (Fig. 7a) in the PDF represents distances from the second and third nearest neighbor distances. At higher r values there are 5 more features in the PDF representing larger distances. These peaks are all spaced between 0.22–0.25 nm, suggesting a regular pattern in the way the polyhedra are arranged relative to each other. At longer length scales the atomic positions are no longer correlated. Figure 7(b) shows expanded view of the first peak.

**Figure 8:**
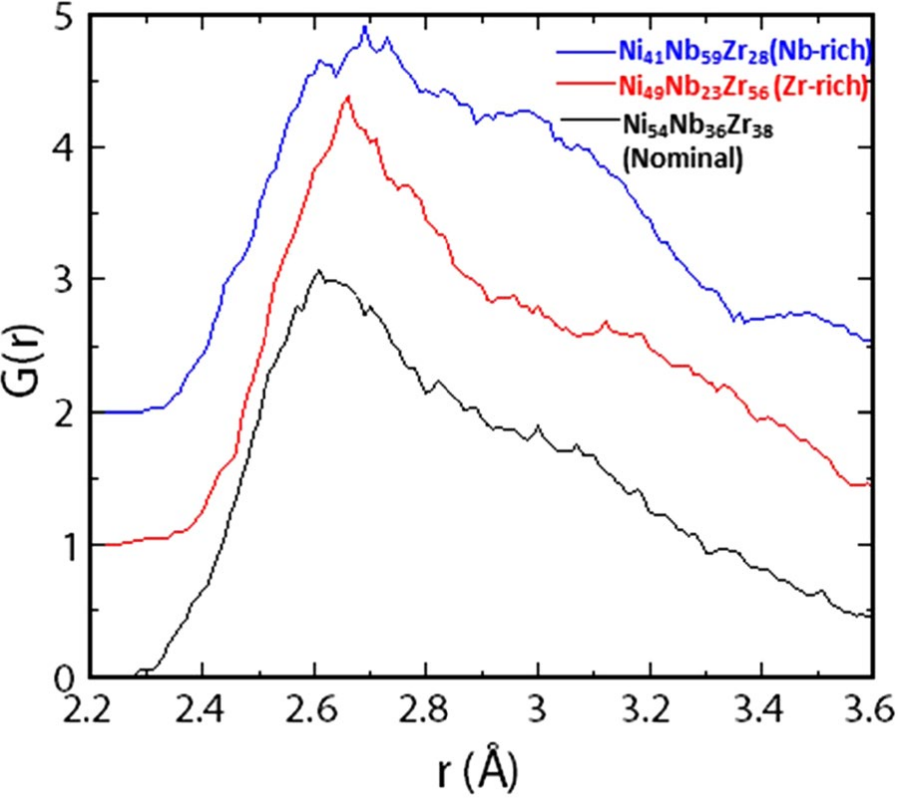
Radial distribution function (RDF) optimizing the structure for nominal composition (Ni_54_Nb_36_Zr_38_), Zr-rich region (Ni_49_Nb_23_Zr_56_) and Nb-rich region (Ni_41_Nb_59_Zr_28_) by black, red and blue color respectively.

We also obtained interatomic distances from radial distribution function (RDF) optimizing the structure for nominal composition (Ni_54_Nb_36_Zr_38_), Zr-rich region (Ni_49_Nb_23_Zr_56_) and Nb-rich region (Ni_41_Nb_59_Zr_28_) compositions from the DFT-MD simulations, as showed in the Fig. 8. The nominal composition data match with neutron total scattering experimental values. It is interesting to observe that in Nb and Zr rich region the peaks shifted on the right side, concluding Ni atom bonded with more number of Nb and Zr than the nominal composition. In the Nb-rich region (Ni_41_Nb_59_Zr_28_), second peak of Nb-Nb distances are more prominent than other two compositions as expected. The data represented here in the Table 5 is only first atomic shell which is less than 0.360 nm observed from both experimental and simulated values. Due to smaller system consideration, the long-range order is not calculated here.

**Table 5:** The interatomic distances from Neutron Scattering and DFT-MD simulation, note that the Ni-Nb and Ni-Zr distances could not be measured accurately.

Element Pair	Ni-Ni	Nb-Nb	Zr-Zr	Ni-Nb	Ni-Zr	Nb-Zr
Ni_42_Nb_28_Zr_30_ (HIPD)	~0.240–0.270	~0.295	~0.324	Slightly larger than 0.268	Slightly larger than 0.268	~0.324
Ni_54_Nb_36_Zr_38_ (DFT –MD)	~0.262	~0.296	~0.325			
Ni_49_Nb_23_Zr_56_(DFT –MD)	~0.265	−0.295	~0.300–0.320			
Ni_41_Nb_59_Zr_28_(DFT –MD)	~0.270	~0.3	~0.340			

Several studies have been done in past few decades in amorphous (Ni_0.60_Nb_0.40_)_70_Zr_30_ as well as Ni-based amorphous ribbon by x-ray diffraction in order to understand interatomic distances between atoms. The summary of the data obtained from EXAFS and XRD-RDF studies are shown in Table 6^36–46^. All the data of nearest neighbor found in the literature study are x-ray data and no reports of neutron studies are available, to best of our knowledge.

**Table 6:** The summary of nearest neighbor distance measurement from previous studies.

References	Year	Alloy	Ni-Ni	Ni-Zr	Zr-Ni	Zr-Zr	Nb-Nb	Ni-Nb	Nb-Zr	Nb-Ni	Ni-Zb
			r (nm)	r (nm)	r (nm)	r (nm)	r (nm)	r (nm)	r (nm)	r (nm)	r (nm)
Frahm *et al*.^36^	1984	Ni_24.1_Zr_75.9_	—	0.262	0.262	0.317	—	—	—	—	—
Frahm *et al*.^36^	1984	Ni_33.3_Zr_66.7_	—	0.262	0.262	0.318	—	—	—	—	—
Frahm *et al*.^36^	1984	Ni_36.5_Zr_63.5_	—	0.262	0.262	0.32	—	—	—	—	—
Lee *et al*.^37^	1984	Ni_35_Zr_65_	0.266	0.269	0.269	0.315	—	—	—	—	—
Lee *et al*.^37^	1985	Ni_35_Zr_65_	0.266	0.269	0.269	0.315	—	—	—	—	—
Frahm *et al*.^38^	1989	DSC Ni_36.5_Zr_63.5_	—	0.263	0.263	0.321	—	—	—	—	—
Lima *et al*.^39^	1988	Ni_25_Zr_75_	—	0.276	0.276	3.23	—	—	—	—	—
Paul *et al*.^40^	1990	Ni_24.1_Zr_75.9_	—	0.27	0.27	0.316	—	—	—	—	—
Paul *et al*.^40^	1990	Ni_33.3_Zr_66.7_	—	0.27	0.27	0.316	—	—	—	—	—
Paul *et al*.^40^	1990	Ni_36.5_Zr_63.5_	—	0.27	0.27	0.316	—	—	—	—	—
Paul *et al*.^40^	1990	DSC Ni_36.5_Zr_63.5_	—	0.27	0.27	0.324	—	—	—	—	—
Babanov *et al*.^41^	1995	Ni_67_Zr_33_	0.249	0.272	—	—	—	—	—	—	—
Lima *et al*.^42^	2003	a-NiZr_2_	0.267	0.267	0.267	0.321	—	—	—	—	—
Yamuara *et al*.^10^	2005	Ni_60_Nb_40_	0.247	—	—	—	0.294	0.266	—	0.266	—
Yamuara *et al*.^10^	2005	(Ni_0.6_Nb_0.4_)_70_Zr_30_	0.25	0.266	0.266	0.32	0.292	0.268	0.311	0.268	0.311
Sakurai *et al*.^43^	2005	(Ni_0.6_Nb_0.4_)_70_Zr_30_		0.263		0.316	—	—	—	—	—
Yamuara *et al*.^10^	2005	(Ni_0.6_Nb_0.4_)_50_Zr_50_	0.25	0.266	0.266	0.32	0.292	0.269	0.311	0.269	0.311
Sakurai *et al*.^43^	2005	(Ni_0.6_Nb_0.4_)_50_Zr_50_	—	0.264		0.316	—	—	—	—	—
Saida *et al*.^44^	2007	Ni_30_Zr_70_	0.248	—	0.27	0.319	—	—	—	—	—
Oji *et al*.^12^	2009	(Ni_0.6_Nb_0.4_)_70_Zr_30_	—	—	0.263	0.324			0.324	0.255	—
Oji *et al*.^12^	2009	(Ni_0.6_Nb_0.4_)_60_Zr_40_	—	—	0.263	0.325		—	0.325	0.254	—
Tian *et al*.^45^	2012	Ni_62.5_Nb_37.5_	2.48	—	—	—	3.05	2.63	—	5.5	—
Yang *et al*.^46^	2013	Ni_60_Nb_40_	0.273	—	—	—	0.282	0.28	—	—	—
Yang *et al*.^46^	2013	Ni_61_Nb_39_	0.273	—	—	—	0.282	0.279	—	—	—
Yang *et al*.^46^	2013	Ni_62_Nb_38_	0.273	—	—	—	0.281	0.279	—	—	—
Yang *et al*.^46^	2013	Ni_63_Nb_37_	0.272	—	—	—	0.282	0.278	—	—	—
Yang *et al*.^46^	2013	Ni_64_Nb_36_	0.273	—	—	—	0.281	0.279	—	—	—

The advantage of PDF over XAFS is that later is limited to the first 1–3 coordination spheres, whereas we can access much longer interatomic distances with the PDF. The amorphous structure of (Ni_0.60_Nb_0.40_)_70_Zr_30_ has order up to ~1.8 nanometer, but is completely disordered when viewed at longer length scales. The combination of APT, PDF and DFT-MD simulations comprehensively suggest that there are large clusters with 2–5 nm in diameter, but there is not necessarily structural coherence across the entire length of the cluster.

The cluster formation with in the amorphous material affects permeation property significantly. In general, the permeation depends on both solubility and diffusivity of the material. According to Yamuara’s *et al*.^10^ study, the solubility Ni-Zr alloy increases with the addition of Nb in the system due to change in local atomic structure. In this study of (Ni_0.6_Nb_0.4_)_70_Zr_30_ metallic glass, Nb interconnected icosahedra cluster region appears with Zr clusters, which helps in hydrogen solubility. Fukuhara *et al*.^32^ suggested that at lower hydrogen concentration, the hydrogen atom localizes to a site between Ni atoms that belong to different icosahedra. However, if the hydrogen content is high, hydrogen atom goes inside tetrahedral sites of distorted Zr and Nb and enlarges the icosahedra. There are several experimental and computational studies^36,37^ reveal volume expansion of the alloy due to hydrogenation. The APT and DFT-MD studies show Ni-based alloys have Nb- and Zr- rich interconnected icosahedra clusters throughout the matrix. Hydrogen insertion causes increase in the cluster size due to enlargement of these icosahedra that creates path for hydrogen to pass through the membrane. It will be interesting to observe how the cluster size affects the permeation property of these Ni-Nb-Zr alloy membranes. These studies are in progress

## Conclusions

Cluster formation within the matrix of Ni-rich amorphous alloy is shown unambiguously by atom probe tomography and DFT-MD simulation. Specifically, a Nb-rich phase of Ni_32_Nb_46_Zr_22_ (cluster size ~4-5 nm), and a Zr-rich phase of Ni_39_Nb_18_Zr_44_ (cluster size ~2–3 nm) are embedded in the Ni-rich ternary Ni_42_Nb_28_Zr_30_ amorphous alloy matrix. The DFT-MD simulation reveals that the Nb-rich phase composed of 7 icosahedra and Zr-rich phase composed of 3 icosahedra. This study reveals Nb-rich interconnected full icosahedra restrain long range crystallization and increases stability of the metallic glass. The HIPD PDF data show that the short range order is indeed present, and extends up to ~1.8 nm. This means that there is some order beyond the length scale of a single icosahedral unit but there is generally no structural order across the entire length of a cluster. The interatomic distances calculated from experiments and DFT-MD simulations agree very well, even though the cooling rate in simulations is much higher than experiments. In conclusion, these studies shed light on probable hydrogen permeation mechanisms, structural and dynamic properties of amorphous membranes.

## Methods

High purity nickel (35.1 wt.%), niobium (33.3 wt.%) and zirconium (31.6 wt.%) powder particles were mixed and arc-melted to produce alloy buttons of Ni_42_Nb_28_Zr_30_ at the DOE AMES Laboratory in Iowa. The fast-quenched ribbon, usually designated as, (Ni_0.60_Nb_0.40_)_70_Zr_30_, was fabricated by a melt-spinning under argon atmosphere at CSIRO laboratory, Brisbane, Australia. The cooling rate for fabrication of this ternary ribbon was ~10^6^ K/s. X-ray diffraction analysis performed on the ribbon (~50 μm thick) confirmed the amorphous nature of ribbon.

### Atom Probe Tomography (APT)

A focused-ion beam (FIB) instrument is used to prepare needle shaped specimens for atom probe tomography (APT). Sections of ~30 μm long lift-out volume are extracted from the fast-quenched surface of a melt spun ribbon and mounted on the top of standard APT microtip array tips, and ion milled to form a needle with a sharp tip whose radius of curvature is ~30–50 nm. The samples were then transferred to a Cameca LEAP 4000X Si for APT analysis. APT can achieve a spatial resolution of better than 0.3 nm in three dimensions, and high analytical sensitivity of ~1 atomic parts per million (at.ppm) in reconstructed sample volumes of typically 106 nm3 (100 nm × 100 nm × 100 nm). The detection efficiency of the LEAP 4000X Si is approximately 50%. In spite of these limitations, APT is the best probe available for studying local compositions in materials on a sub-nm scale in three dimensions. For the APT studies, a pulsed laser beam is utilized, with a wavelength of 355 nm (ultraviolet) and 20 pJ pulse energies to activate atom-by-atom field evaporation, with a pulse repetition frequency of 500 kHz and a specimen stage temperature of 30 K. More details on the APT method can found in refs^47–49^.

### Neutron Total Scattering

The ribbon of (Ni_0.60_Nb_0.40_)_70_Zr_30_ was loaded in the HIPD instrument at the Lujan Neutron Scattering Center of Los Alamos National Laboratory and neutron total scattering data was collected. The pair distribution function, G(r), was generated from S(Q) using a Q_max_ of 25 Å^−1^ with the program PDFgetN; details can be found in the ref.^50^.

### Quantum molecular dynamics simulation

The atomic structures and interatomic distances in (Ni_0.60_Nb_0.40_)_70_Zr_30_ alloy membrane are calculated by DFT and DFT-based molecular dynamics (DFT-MD) simulations. The DFT-MD simulations (or *ab initio* molecular dynamics simulations) use interatomic forces calculated from quantum mechanics. All these calculations are performed using VASP (The Vienna Ab initio Simulation Package) with plane wave basis set^51,52^. The Perdew-Burke-Ernzerhof (PBE) Functional is applied for the generalized gradient approximation (GGA) exchange-correlation functional. In addition, the full potential projector augmented-wave (PAW) method was adopted for the core-valence interaction. To optimize the local structure of amorphous alloy, the energy cutoff for plane wave expansions was set to 600 eV. For supercell calculations, reciprocal space was sampled using the Γ-centered Monkhorst-Pack scheme with gamma point only. For geometric optimization, the force convergence criterion is considered as 10^−3^ eV/A° and electronic self-consistent field (SCF) convergence is 10^−6^ eV.

The DFT-MD simulation steps are discussed below. We started with the composition Ni_54_Nb_36_Zr_38_ in which 128 atoms were randomly placed in the supercell with the cell length of 12.86 Å along three directions. This leads to a density of 7.79 g/cm^3^, and the other two systems of Zr-rich composition Ni_49_Nb_23_Zr_56_ and Nb-rich composition Ni_41_Nb_59_Zr_28_ are also constructed with the initial density of 7.46 and 7.94 g/cm^3^, respectively. The systems were first heated from room temperature to 3000 K within 2 ps and then equilibrated at 3000 K for 5 ps using NVT ensemble (constant volume, constant temperature and constant number of atoms). Finally the systems are quenched from 3000 K to room temperature over the period of 25 ps with a fixed volume. This leads to a very fast quench rate of 1.08 × 10^14^ K/s. The time step is set to 2 fs in the DFT-MD simulations. The convergence criterion is set to a 1 × 10^−4^ eV energy difference for solving the electronic wave function in DFT-MD simulations. After we obtained the amorphous structures in DFT-MD simulations, we performed geometry relaxation using DFT to relax the internal stress. Then we performed the structural analyses such as RDF and Voronoi analysis^53^ on these relaxed structures. The radial distribution function (RDF) describes how density varies as a function of distance from a reference particle. It is a very useful tool to distinguish the structures between crystal and amorphous materials. The RDF only reveals the average structure information. To characterize the topological short range order, we use the Voronoi tessellation analysis. Here the indices (n3, n4, n5, n6) represent the number of i-edge faces of the polyhedron where the sum of ni gives the coordination number (CN) of the center atom.
